# Apt-Conjugated PDMS-ZnO/Ag-Based Multifunctional Integrated Superhydrophobic Biosensor with High SERS Activity and Photocatalytic Sterilization Performance

**DOI:** 10.3390/ijms25147675

**Published:** 2024-07-12

**Authors:** Sihan Qian, Wenshi Zhao, Rui Guo, Xiaohan Wang, Huasong Dai, Jihui Lang, Naveen Reddy Kadasala, Yuhong Jiang, Yang Liu

**Affiliations:** 1 Key Laboratory of Functional Materials Physics and Chemistry of the Ministry of Education, Jilin Normal University, Changchun 130103, China; q1244234477@163.com (S.Q.); 2405006@jlnu.edu.cn (W.Z.); guorui3626@163.com (R.G.); wxhhh990929@163.com (X.W.); 15948835770@163.com (H.D.); jhlang@jlnu.edu.cn (J.L.); 2 Department of Chemistry, Towson University, Towson, MD 21252, USA; naveenkadasala@gmail.com

**Keywords:** surface-enhanced Raman scattering, aptamer, finite-difference time-domain, photocatalytic sterilization, *Salmonella typhimurium*

## Abstract

Sensitive detection and efficient inactivation of pathogenic bacteria are crucial for halting the spread and reproduction of foodborne pathogenic bacteria. Herein, a novel Apt-modified PDMS-ZnO/Ag multifunctional biosensor has been developed for high-sensitivity surface-enhanced Raman scattering (SERS) detection along with photocatalytic sterilization towards Salmonella typhimurium (*S. typhimurium*). The distribution of the electric field in PDMS-ZnO/Ag with different Ag sputtering times was analyzed using a finite-difference time-domain (FDTD) algorithm. Due to the combined effect of electromagnetic enhancement and chemical enhancement, PDMS-ZnO/Ag exhibited outstanding SERS sensitivity. The limit of detection (LOD) for 4-MBA on the optimal SERS substrate (PZA-40) could be as little as 10^−9^ M. After PZA-40 was modified with the aptamer, the LOD of the PZA-40-Apt biosensor for detecting *S. typhimurium* was only 10 cfu/mL. Additionally, the PZA-40-Apt biosensor could effectively inactivate *S. typhimurium* under visible light irradiation within 10 min, with a bacterial lethality rate (L_b_) of up to 97%. In particular, the PZA-40-Apt biosensor could identify *S. typhimurium* in food samples in addition to having minimal cytotoxicity and powerful biocompatibility. This work provides a multifunctional nanoplatform with broad prospects for selective SERS detection and photocatalytic sterilization of pathogenic bacteria.

## 1. Introduction

For both developed and developing countries, foodborne pathogens pose a major risk to human health and life. They are a significant source of foodborne disease outbreaks [1]. The reason why the foodborne infection accidents are fairly common is that it is hard to prevent the spread of foodborne pathogens. In fact, foodborne pathogenic bacteria can be introduced into the body just through drinking contaminated water or eating contaminated food [2]. The World Health Organization (WHO) estimates that, in 2010 alone, pathogenic bacteria were responsible for about 600 million foodborne diseases and 420,000 deaths globally [3]. Among all foodborne pathogenic bacteria, the food poisoning caused by salmonella with more than 2500 serotypes often tops the lists of foodborne pathogens [4,5]. In particular, *Salmonella typhimurium* (*S. typhimurium*), with high virulence, multidrug-resistance, high transmissibility and a wide host range, is responsible for the highest hospitalization rates and it has already been proven that *S. typhimurium* is linked to outbreaks of infectious diseases in animals and humans worldwide [6]. It has been shown that even ingestion of 50 to 70 CFU of *S. typhimurium* can cause bacterial infection [7]. Symptomatic illness infected with *S. typhimurium* is manifested by diarrhea, fever and stomach cramps [8]. If not treated in time, *S. typhimurium* may traverse from the gastrointestinal tract into the bloodstream and cause acute gastroenteritis, bacteremia, septicemia and even death [9]. The design and construction of a new multifunctional biosensor to achieve high-sensitivity detection as well as efficient inactivation for *S. typhimurium* is therefore of great importance.

At present, culture methods, gas chromatography–mass spectrometry (GC-MS), electrochemical sensing, polymerase chain reaction (PCR) and loop-mediated isothermal amplification (LAMP) are the most commonly used detection methods for *S. typhimurium* [10,11,12,13]. Although these techniques have excellent sensitivity and specificity, they have a number of limitations, including the requirement for professional personnel and expensive instruments, the time-consuming sample pretreatments, and the potential for contamination by other bacteria during the detection process [14]. These limitations make it difficult to put these techniques into practice. Because of its great sensitivity, ease of use, outstanding stability, fast detection speed and robust selectivity, surface-enhanced Raman scattering (SERS) holds great promise for identifying and detecting foodborne pathogens [15]. Through the amplification of Raman signals from molecules adsorbed on or close to particular substrates like metal nanostructures, SERS can provide molecular fingerprints of analytes. High SERS performance generally depends on two enhancement mechanisms: the electromagnetic mechanism (EM) and the chemical mechanism (CM) [16]. Among these, EM is primarily caused by the localized surface plasmon resonance (LSPR) for noble metal nanostructures. This is commonly considered to be the main reason for SERS [17,18]. The most widely utilized SERS active substrates are Au and Ag among the developed SERS substrates [19,20]. Ag nanocrystals are the first choice for SERS active substrates because they have a stronger SERS enhancement effect than Au nanocrystals [21]. Although EM is the primary factor of the enhancement of SERS, the contribution of CM should not be undervalued. It is possible to produce chemical enhancement by combining semiconductors (such as SiO_2_, TiO_2_, Fe_2_O_3_, and ZnO) and noble metal Ag nanocrystals. This combination can facilitate charge transfer (CT) between the noble metal and semiconductor, speeding up CT from SERS substrates to targeted molecules and thereby obtaining the synergistic enhancement of SERS signals [22]. Another advantage of the combined use of semiconductors and noble metal Ag nanocrystals is to address the drawbacks of Ag nanocrystals, such as easy aggregation and instability [23]. Among the semiconductor–noble metal nanocomposites mentioned above, the hybrids of ZnO and Ag have emerged as leaders due to their superior SERS signals. In particular, the rod-shaped ZnO nanocrystals with a large specific surface area have received special attention, which will help metallic Ag nanocrystals bind to the surfaces of ZnO nanocrystals to produce more “hot spots” [24]. Additionally, rod-like ZnO/Ag nanocomposites can boost the production of the photogenerated electrons (e^−^) due to the difference of the Fermi lever between ZnO and Ag, and the e^−^ accumulated in the interfaces of rod-like ZnO/Ag nanocomposites can greatly strengthen the local electromagnetic field, aiding an improving Raman signal of the targeted molecules [25].

Massive effort has been put into designing and fabricating several types of SERS substrates during the past few decades, primarily including colloidal SERS substrates and solid SERS substrates [26]. A large density of SERS-active hot spots can be produced by combining colloidal SERS substrates of different sizes and shapes with certain salts or solvents, significantly improving the plasmonic electromagnetic field [27]. However, because it is challenging to regulate the degree of aggregation of colloidal SERS substrates, the repeatability and analytical sensitivity of this approach are not optimal [28]. The solid SERS substrates, by contrast, exhibit both high reproducibility and sensitivity of SERS signals. Unfortunately, the conventional solid SERS substrates are often made of glass plates, silicon wafers, and quartz slides, which are brittle and stiff and are not suited for directly detecting analytes that are affixed to curved surfaces, significantly restricting the practical application of SERS technology [29]. Flexible solid SERS substrates, on the other hand, have attracted considerable attention for their applications in real-time monitoring of chemical and biological samples as well as on-site Raman measurements [30]. The flexible material polydimethylsiloxane (PDMS) is safe to use, transparent optically and chemically stable, making it a promising candidate for developing a novel flexible hybrid SERS substrate [31,32]. The other significant advantage of PDMS as the ideal matrix of plasmonic nanoparticles is its naturally hydrophobic surface, which can not only prevent the analyte solution from randomly diffusing and achieve the enrichment effect, but also hinder the coffee-ring effect, thus improving the detection limit in SERS analysis [33]. The introduction of recognition molecules with high affinity and specificity is also crucial in order to precisely identify and bind to target bacteria in complex environments [34]. Aptamers (Apt) offer several benefits over other recognition components (e.g., antibodies) that bind to target molecules specifically, including ease of operation, low production costs, high stability, improved measurement flexibility and specific recognition capability [35,36]. As a result, PDMS-ZnO/Ag modified with aptamers is anticipated to enable sensitive detection of target molecules, making it suitable for the requirements of real-world SERS applications.

As well as sensitively detecting *S. typhimurium*, it is also urgent to effectively inactivate bacteria. In recent years, photocatalytic sterilization has gained recognition as a simple, environmentally friendly, efficient and low-cost method for eliminating bacteria [37]. Under visible light irradiation, photo-induced charge carriers are generated, which can interact with O_2_ and H_2_O to create reactive oxygen species (ROS) like hydroxyl radicals (·OH) as well as reactive oxygen ions (·O^2−^) which are free radicals [38,39]. Given the potent oxidizing abilities of ROS, they have the capability to effectively destroy the bacterial cell membrane as well as wall structure through the oxidation process, leading to the outflow of metabolites from internal cell substances and thereby achieving the desired sterilization effect [40]. The combination of ZnO and Ag, fortunately, not only makes it possible for SERS detection with extreme sensitivity, but also works incredibly well as a photocatalytic sterilization agent [41]. It should be noted that although ZnO nanoparticles have weak antibacterial activity, modification with the noble metal Ag can generate significant antibacterial activity [42]. On one hand, due to work function and band alignment differences between the noble metal and semiconductor, a Schottky barrier will form at their interface. This enhances photocatalytic activities by successfully promoting e^−^ and holes (h^+^) to separate [43]. On the other hand, Ag nanocrystals are considered to have a wide range of bactericidal effects, and, even at very low concentrations, Ag has strong sterilization activity [44]. Therefore, combining the bactericidal activity of ZnO/Ag nanohybrids with photocatalytic sterilization is a promising strategy to achieve a dual sterilization effect and thus trigger bacterial death [45]. In addition, the addition of aptamers and PDMS in the ZnO/Ag nanocomposites endows them with exceptional selective recognition/targeting ability and enrichment abilities, thereby inactivating them more effectively [46,47]. But, up until now, the majority of research only focused on constructing a single-function biological system that could either detect or eliminate *S. typhimurium*. Little research has been devoted to the combination of high sensitivity detection and bacterial inactivation to prevent *S. typhimurium* infection.

Herein, a novel multifunctional Apt-conjugated PDMS-ZnO/Ag biosensor was designed and constructed to enable ultrasensitive SERS detection and photocatalytic sterilization of *S. typhimurium* in intricate samples. The biosensor was composed of ZnO nanorods, Ag nanocrystals and a flexible PDMS substrate, and then was functionalized with aptamers to specifically bind with *S. typhimurium*. For the comparison of the SERS activity of PDMS-Ag and PDMS-ZnO/Ag, 4-mercaptobenzoic acid (4-MBA) was selected as the Raman reporter molecule. Additionally, employing a finite-difference time-domain (FDTD) approach, the distribution of “hot spots” was simulated and the influence of Ag sputtering time on SERS signal intensity was discussed. By analyzing the correlation between interparticle gaps and SERS enhancement, a potential SERS enhancement mechanism was proposed and the PZA-40 SERS substrate with optimal SERS enhancement performance was achieved. The superhydrophobic property and SERS activity in practical applications of a PZA-40-Apt biosensor were also investigated. In addition, the PZA-40-Apt biosensor successfully inactivated *S. typhimurium* when exposed to visible light. The mechanism behind the deactivation of *S. typhimurium* by PZA-40-Apt was also revealed. This study not only elucidates the SERS enhancement mechanism and bacterial inactivation mechanism of the multifunctional biosensor, but also provides an ideal platform for sensitive detection and efficient inactivation of bacteria.

## 2. Results and Discussion

### 2.1. Characterization of PDMS-ZnO/Ag Films with Different Ag Sputtering Times

**Figure 1:**
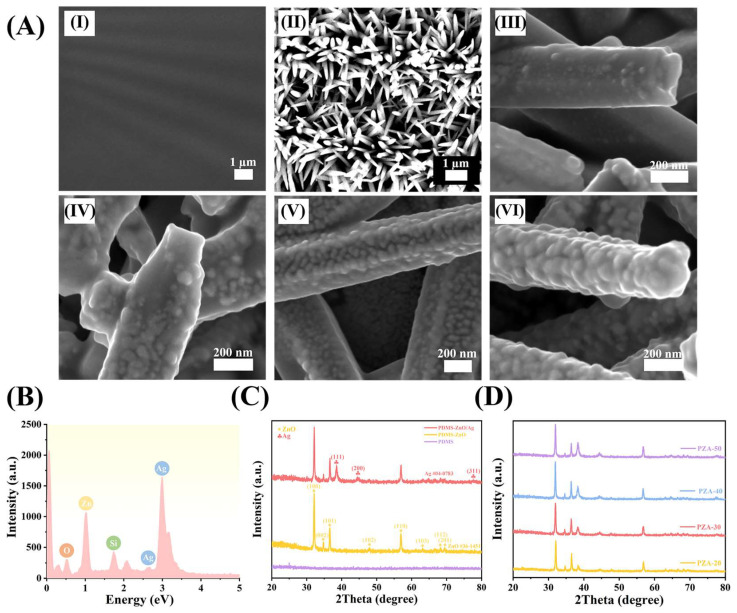
(**A**) SEM images of (I) PDMS, (II) PDMS-ZnO and PDMS-ZnO/Ag with different sputtering times of (III) 20 s, (IV) 30 s, (V) 40 s and (VI) 50 s. (**B**) EDS spectra of PZA-40. XRD patterns of (**C**) PDMS, PDMS-ZnO and PDMS-ZnO/Ag and (**D**) PZA-20, PZA-30, PZA-40 and PZA-50.

SEM and XRD were performed to investigate the morphologies and crystal structures of PDMS, PDMS-ZnO film and PDMS-ZnO/Ag films with various Ag sputtering times (20, 30, 40 and 50 s). Figure 1A(I) shows that the surface of PDMS is very smooth. In addition, the prepared PDMS was subjected to flexibility experiments, as shown in Figure S1, which demonstrated that PDMS has good flexibility. As exhibited in Figure 1A(II), ZnO nanorods with dimensions of roughly 3.5 µm in length and 320 nm in diameter were successfully grown on PDMS film. Ag nanocrystals were observed to be stochastically and homogeneously adsorbed onto the surfaces of ZnO nanorods after being sputtered onto PDMS-ZnO films, as presented in Figure 1A(III,VI). Increasing the Ag sputtering time from 20 to 40 s led to a gradual increase in the amount of Ag nanocrystals on the surfaces of the ZnO nanorods, leading to rougher surfaces. Interestingly, when the Ag sputtering time was further increased to 50 s, the Ag nanocrystals self-aggregated due to their excess introduction. Obviously, this is the cause of the reduced surface roughness of ZnO nanorods. The EDS results presented in Figure 1B confirm that PZA-40 is made up of Zn, O, Si and Ag elements. As for PDMS, no discernible XRD diffraction peaks are observed in Figure 1C. By contrast, the XRD pattern of PDMS-ZnO film has eight diffraction peaks at 32.12°, 34.74°, 36.62°, 47.86°, 56.94°, 63.14°, 68.22° and 69.48°. These peaks correspond to (100), (002), (101), (102), (110), (103), (112) and (201) planes of ZnO with the hexagonal wurtzite structure (JCPDS card No. 36-1451) [48]. When Ag nanocrystals were deposited on the PDMS-ZnO film surfaces, three further diffraction peaks were observed at 38.56°, 44.46° and 77.72°. These peaks were assigned to (111), (200) and (311) planes of face-centered cubic Ag (JCPDS card No. 04-0783), which further confirms the successful preparation of PDMS-ZnO/Ag films [23]. All three samples exhibited no impurity phases in their XRD patterns. In the meanwhile, PDMS-ZnO/Ag films with various Ag sputtering times were compared by means of their XRD patterns. Figure 1D shows that the strength of the Ag diffraction peak increases as Ag sputtering time increases from 20 s to 50 s. This is consistent with SEM observations and suggests that more Ag nanocrystals are accumulated on the PDMS-ZnO/Ag.

### 2.2. XPS Analysis of PDMS-ZnO/Ag Films

**Figure 2:**
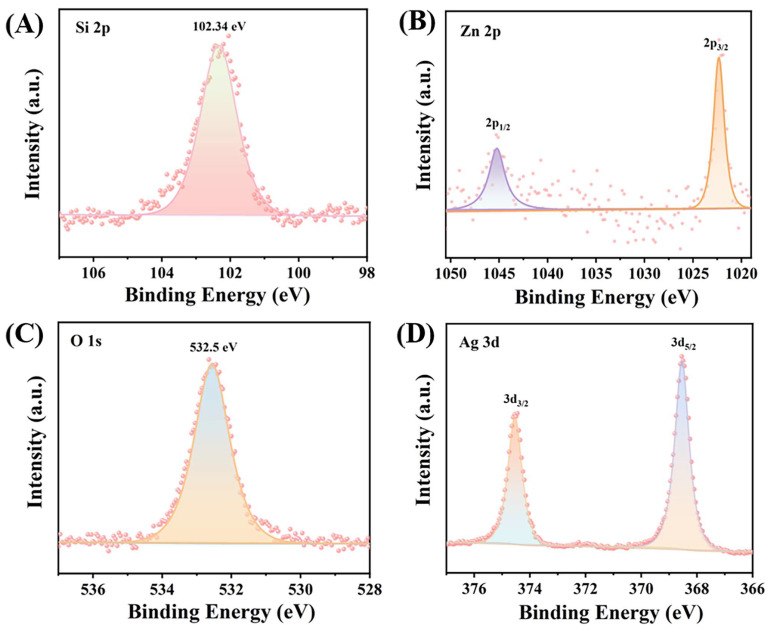
XPS spectra of PZA-40: (**A**) Si 2p, (**B**) Zn 2p, (**C**) O 1s and (**D**) Ag 3d.

The elemental compositions, chemical states and electromigration processes of PDMS-ZnO/Ag films were studied using XPS. For charge correction, the C 1s peak at 284.8 eV was employed. The Si, O, Zn and Ag elements are found in the XPS full survey spectrum of PZA-40, as shown in Figure S2A. Based on the Gauss fitting, the Si 2p XPS spectrum of PZA-40 in Figure 2A shows that (Si(CH_3_)_2_-O-)n of PDMS is responsible for the peak of 102.34 eV [49]. The XPS spectra for Zn 2p (Figure 2B) show two contributions, namely Zn 2p_1/2_ and Zn 2p_3/2_ (arising from a spin-orbit splitting), located at 1045.22 and 1022.32 eV, respectively. These contributions can be assigned to ZnO [50]. The O 1s XPS spectrum in Figure 2C reveals a solitary peak at 532.5 eV, which is linked to the photoemission in O^2−^ of Zn-O bonding in ZnO [51]. Figure 2D reveals two peaks for Ag 3d in the binding energies (BEs) of 374.54 eV (Ag 3d_3/2_) and 368.54 eV (Ag 3d_5/2_) with a spin-orbit splitting of around 6 eV, suggesting the presence of a Ag^0^ state in PZA-40 [52,53]. The results of XPS are more compelling evidence of the successful preparation of a PDMS-ZnO/Ag film. Interestingly, as Figure S2B illustrates, the Zn 2p BEs of PDMS-ZnO/Ag migrate in a positive direction when compared to PDMS-ZnO. This shows a reduction in electron density resulting from CT from the conduction band (CB) of ZnO to the Fermi level of Ag [23]. As for PZA-40, the displacement of BEs reaches a maximum, signifying the highest level of electron transport between Ag and ZnO. The shift in the BEs of PZA-40 is the maximum and peaks of Ag 3d_5/2_ and Ag 3d_3/2_ show a negative shift when compared to the BEs of PDMS-Ag, demonstrating the increase in electron density on the Ag level (Figure S2C). The BEs shifts of Zn 2p and Ag 3d occur simultaneously and in opposite directions, indicating electron transfer from ZnO to Ag by their interaction. This process facilitates the detachment of photoexcited e^−^-h^+^ pairs and inhibits e^−^-h^+^ recombination.

### 2.3. SERS Activity and Mechanism of PDMS-ZnO/Ag Films with Different Ag Sputtering Times

**Figure 3:**
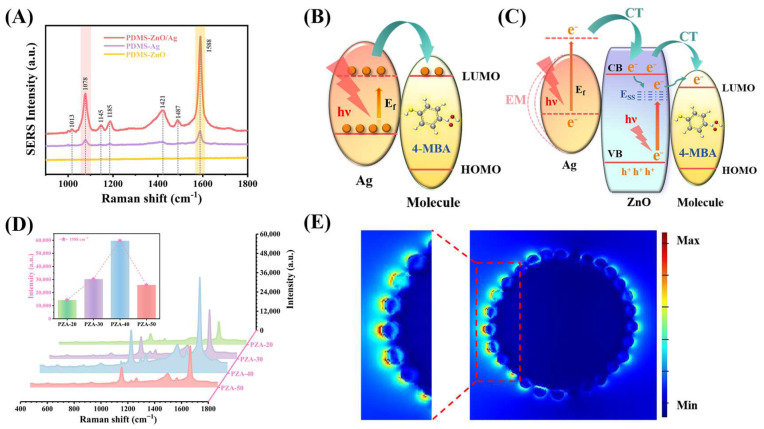
SERS spectra of (**A**) 4-MBA on PDMS-ZnO, PDMS-Ag and PDMS-ZnO/Ag. Diagrams of the CT process of between Ag and 4-MBA molecules (**B**) and among Ag, ZnO and 4-MBA molecules (**C**). (**D**) SERS spectra of 4-MBA adsorbed on PZA-20, PZA-30, PZA-40 and PZA-50. The inset of (**D**) was the corresponding variation in SERS intensity at 1588 cm^−1^. (**E**) FDTD method simulation of the electric field distribution of PZA-40.

SERS spectra of 4-MBA (10^−5^ M) adsorbed on PDMS-ZnO, PDMS-Ag and PDMS-ZnO/Ag films were recorded as presented in Figure 3A. Meanwhile, the SERS spectrum of 4-MBA (10^−5^ M) adsorbed on PDMS is shown in Figure S3. The purpose was to compare and assess the SERS performance of PDMS-ZnO/Ag. One can observe that there is virtually no Raman signal for 4-MBA which is adsorbed on PDMS and PDMS-ZnO. However, PDMS-Ag can produce a distinct and identifiable 4-MBA characteristic peak. This is because of the LSPR effect caused by Ag. The Raman spectra for pure 4-MBA molecules are displayed in Figure S4, while Table S1 provides a comprehensive list of SERS band assignments of 4-MBA molecules [54,55]. After Ag nanocrystals are deposited on PDMS-ZnO films, PDMS-ZnO/Ag films have 3.3-fold enhancement in comparison to PDMS-Ag using 1588 cm^−1^ as the calibration. Although the major contributor to SERS enhancement is generally considered to be the EM mechanism, the chemical enhancement mechanism may be more important than the electromagnetic enhancement mechanism, given that the difference between the two samples is the existence of ZnO. To explain the reasons for the significant enhancement in SERS activity, we proposed a potential mechanistic diagram of the CT process, as illustrated in Figure 3B,C. After Ag nanocrystals are excited through LSPR adsorption, the e^−^ can go straight to the lowest unoccupied molecular orbital (LUMO) energy level in the 4-MBA from the Fermi energy level of Ag (Figure 3B) [56,57]. As depicted in Figure S5, in Ag/ZnO/4-MBA system, the band gap energy of ZnO is reduced to 2.24 eV from 3.12 eV owing to the LSPR effect of Ag. ZnO is essential to the electron transfer between Ag and 4-MBA, as demonstrated in Figure 3C. The introduction of photoexcited e^−^ into the CB of ZnO may lead to their vibrational relaxation to the surface-state energy level (E_SS_), followed by their transfer to LUMO of 4-MBA. The CB and E_SS_ of ZnO act as a “bridge” between Ag nanocrystals (donor) and 4-MBA (receptor) to create the so-called “donor–bridge–acceptor” CT mode [58]. An inadequate amount of Ag nanocrystals can not produce a sufficient amount of photoexcited e^−^. On the other hand, an excessive amount of Ag nanocrystals will coat the ZnO nanorods’ surface, resulting in inadequate absorption of light [22]. Thus, we designed and prepared PDMS-ZnO/Ag films with different nanogaps between Ag nanocrystals by adjusting the sputtering time of Ag. Figure 3D illustrates the SERS spectra of 4-MBA adsorbed on PZA-20, PZA-30, PZA-40 and PZA-50. A comparison of SERS intensity of 4-MBA at 1588 cm^−1^ is also shown. It is clear that by increasing the Ag sputtering time from 20 to 40 s, the SERS intensity of 4-MBA progressively increases. However, it drops abnormally as the sputtering time is increased to 50 s. Because the only distinction between the four samples is the added amount of Ag, the major contribution to SERS enhancement should be ascribed to the EM mechanism originating from the excitation of LSPR on Ag nanostructures [59]. Strong electromagnetic fields, called “hot spots”, are created in/between neighboring noble metal nanocrystals as a result of the coupling effect between them, which can considerably enhance the SERS signals of target analytes [60,61]. It makes sense that a much larger quantity of hot spots will result in stronger SERS enhancement, since the increase in SERS signal strength is a result of repeated collisions between photons excited by the incoming light and the noble metal nanocrystals [62]. The FDTD method was utilized to verify the aforementioned theory. By using a normal incident light source polarized along the x-axis at a wavelength of 514 nm, the FDTD approach was used to model and investigate the electromagnetic field intensity dispersion for PZA-30, PZA-40 and PZA-50 under periodic boundary circumstances. The hot spots are mainly located on the tops of Ag nanocrystals as well as in the gaps among Ag nanocrystals, as seen in Figure 3E and Figure S6. PZA-40 has more Ag nanocrystals on its surface than PZA-30, so it is inevitable that PZA-40 has a better SERS performance compared to PZA-30. By contrast, even while the amount of Ag nanocrystals on the surface of PZA-50 remains higher than PZA-40, the superfluous addition of Ag nanocrystals leads to agglomeration, thus reducing the total amount of hot spots, which, as a result, causes an unexpected decrease in the SERS signal.

### 2.4. SERS Sensitivity and Uniformity of PDMS-ZnO/Ag Film

**Figure 4:**
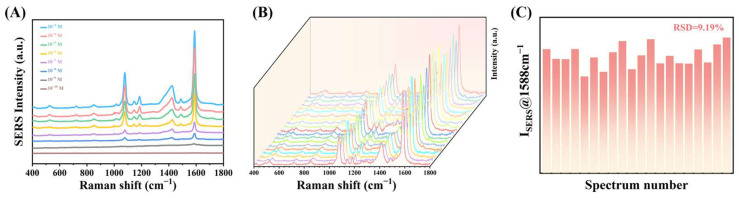
(**A**) SERS spectra of 4-MBA (10^−3^–10^−10^ M) on PZA-40 SERS substrate. (**B**) SERS spectra of 4-MBA recorded from 20 random spots on PZA-40 and (**C**) the corresponding SERS intensities of 4-MBA at 1588 cm^−1^.

The sensitivity and uniformity of PDMS-ZnO/Ag film were also evaluated. The SERS spectra of 4-MBA, which was absorbed on PZA-40 at concentrations ranging from 10^−3^ to 10^−10^ M, was recorded. Figure 4A indicates that the SERS signal strength of 4-MBA falls as the concentration drops, with the limit of detection (LOD) of 10^−9^ M. Compared to other developed SERS substrates, our suggested SERS platform has the greatest SERS sensitivity, as indicated in Table 1 [63,64,65,66]. The SERS enhancement factor (EF) was computed in detail and is available in Supplementary Materials to assess the SERS substrate performance in more depth. The EF value of PZA-40 is as high as 3.3 × 10^5^, while the EF values for PDMS-Ag and PDMS-ZnO are only 1.0 × 10^5^ and 6.0 × 10^3^, respectively. For the SERS substrate, good homogeneity is just as crucial as high SERS sensitivity. SERS spectra of 4-MBA (10^−5^ M) were obtained by picking at random 20 different spots on PZA-40 to assess the uniformity of the PZA-40 SERS substrate. It is apparent from Figure 4B,C that the relative standard deviation (RSD) value of the SERS signal strength is lower than 10% at 1588 cm^−1^ and the signal strength remains nearly constant. The great sensitivity and excellent uniformity of our SERS platform are demonstrated by all of the above results.

### 2.5. Ultrasensitive Detection of S. typhimurium by SERS Multifunctional Biosensor with Enhanced Stability

**Figure 5:**
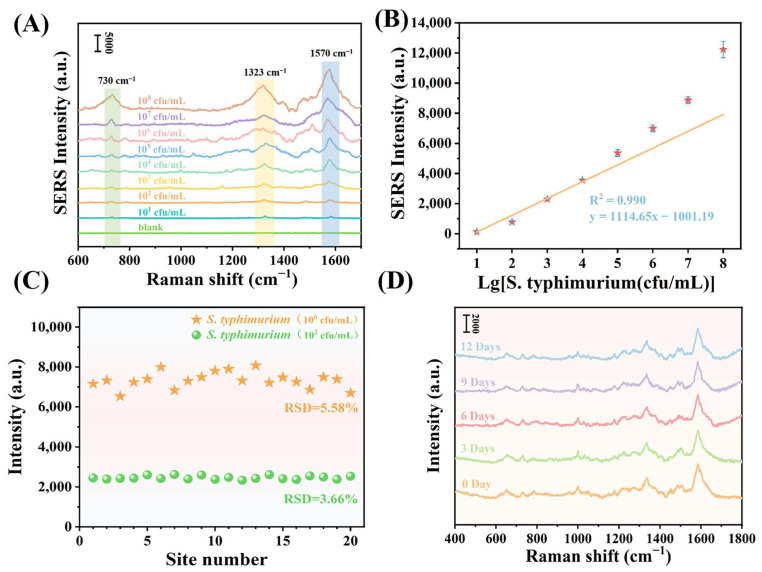
(**A**) SERS spectra for *S. typhimurium* at concentrations ranging from 10^1^ to 10^8^ cfu/mL and (**B**) the corresponding plot of SERS intensity at 1570 cm^−1^ and logarithmic concentration of *S. typhimurium*. (**C**) SERS intensities of the peaks at 1570 cm^−1^ collected from 20 random spots on SERS substrates containing the suspension of *S. typhimurium* with a concentration of 10^6^ and 10^3^ cfu/mL. (**D**) SERS spectra of *S. typhimurium* based on PZA-40-Apt biosensor stored for different days.

The PZA-40-Apt biosensor was prepared by modifying the surface of PZA-40 with aptamers to create a detection platform for bacterial recognition. Figure S7 provides a comparison of the UV-Vis absorption spectra of supernatants before and after conjugating aptamers with PZA-40. Both UV-Vis spectra of supernatants show a distinctive peak at 260 nm. This peak is assigned to the C=C-C=C conjugated double bond of purine and pyrimidine bases in aptamers [67]. Following aptamer modification of PZA-40, the absorbance peak of supernatants at 260 nm is significantly lower than it is prior to aptamer modification, which indicates that the aptamer is successfully immobilized on PZA-40 surface [68]. As observed in Figure S8, a Raman spectrometer captured the SERS spectrum of *S. typhimurium* (10^7^ cfu/mL) based on a PZA-40-Apt multifunctional biosensor. *S. typhimurium* has three distinct Raman peaks at 730, 1323 and 1570 cm^−1^. The adenine ring mode is the cause for the peak at 730 cm^−1^. Peaks at 1323 and 1570 cm^−1^ can be assigned to the presence of polyadenine and lipids [69]. The SERS spectra of *S. typhimurium* at various concentrations ranging from 10 to 10^8^ cfu/mL were gathered in optimal experimental circumstances, as depicted in Figure 5A. It is evident that the PZA-40-Apt biosensor is capable of detecting *S. typhimurium* at a low LOD of 10 cfu/mL. Quantitative analysis for *S. typhimurium* was performed using the SERS peak at 1570 cm^−1^. It was found that the natural logarithm of *S. typhimurium* concentration and the SERS intensity have a linear relationship. With a correlation coefficient (R^2^) of 0.990, the linear regression equation is y = 1114.65 x − 1001.19 (Figure 5B). Meanwhile, good uniformity and reproducibility are equally important for the SERS substrate. Twenty points were randomly selected from the suspension of *S. typhimurium* with a concentration of 10^6^ cfu/mL and 10^3^ cfu/mL, respectively, to verify the uniformity and reproducibility of the SERS signals of the SERS platform. As revealed in Figure 5C, the SERS intensity distribution at 1570 cm^−1^ is highly uniform for both concentrations of the *S. typhimurium* suspension with RSD values of 5.58% and 3.66%, respectively. Furthermore, the stability of the PZA-40-Apt biosensor was assessed by measuring the reproducibility of SERS signals. The PZA-40-Apt biosensor was stored at room temperature for 12 days and *S. typhimurium* was tested every 3 days under the same conditions using the PZA-40-Apt biosensor. The SERS intensity of *S. typhimurium* on the PZA-40-Apt biosensor remained stable for 12 days, demonstrating the high stability of the proposed SERS biosensor (Figure 5D).

### 2.6. Superhydrophobicity of PZA-40-Apt Biosensor and SERS Detection of S. typhimurium in Freshly Squeezed Orange Juice 

**Figure 6:**
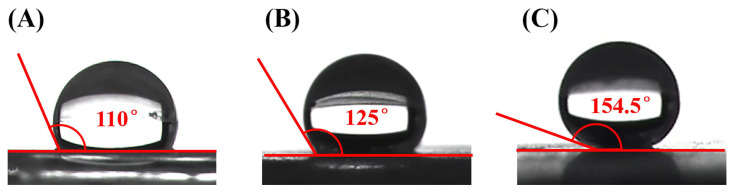
Contact angles of water droplets on surfaces of (**A**) PDMS (**B**) PDMS-ZnO and (**C**) PZA-40-Apt biosensor.

Given that superhydrophobic surfaces with poor water adhesion can raise the concentration of local analytes and hence boost the SERS detection limit in real-life conditions, the wettability of PZA-40-Apt biosensor was evaluated. Figure 6A,B shows that the contact angles between water droplets and PDMS or PDMS-ZnO were measured to be 110° and 125°, respectively. In contrast, as shown in Figure 6C, the water contact angles (WCAs) of the PZA-40-Apt biosensor can be as high as 154.5° (greater than 150°), indicating that the surface of the PZA-40-Apt biosensor has exceptional superhydrophobicity [70,71]. Furthermore, an assessment was conducted on the interfacial behaviors between water droplets and different PDMS substrates. The various PDMS substrates (PDMS, PDMS-ZnO and PZA-40-Apt biosensor) were fixed on the platform. The water droplet firstly moved downwards to increase contact with the PDMS substrates, and then moved upwards to separate itself from the PDMS substrates. As depicted in Figure S9A–C, the water droplet is subjected to extrusion deformation and adheres securely to the surface of PDMS. In comparison, the water droplet can be almost entirely separated from the surfaces of PDMS-ZnO (Figure S9D–F) and the PZA-40-Apt biosensor (Figure S9G–I), which confirms that both PDMS-ZnO and PZA-40-Apt biosensors have low adhesion force to water droplets. 

**Figure 7:**
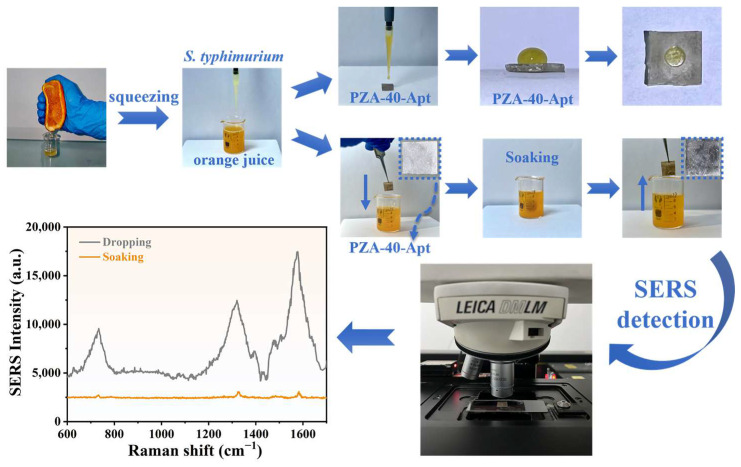
Schematic illustration for SERS detection of *S. typhimurium* in freshly squeezed orange juice samples using PZA-40-Apt biosensor through two methods: “dropping” and “soaking”.

In order to verify the effect of superhydrophobicity on the performance of SERS detection, the PZA-40-Apt biosensor was used for the SERS detection of *S. typhimurium* in freshly squeezed orange juice samples. As shown in Figure 7, a mixture of bacterial solution and orange juice was treated by “dropping” and “soaking” methods, respectively, to compare the effects of the two methods on the SERS signals. Ten µL of orange juice–*S. typhimurium* complex was pipetted onto the surface of the PZA-40-Apt biosensor. Meanwhile, the same biosensor was soaked in the orange juice–*S. typhimurium* complex. Subsequently, the PZA-40-Apt biosensor containing *S. typhimurium* on the surface prepared by “dropping” and “soaking” methods, respectively, was transferred to a glass slide for SERS measurements. By randomly selecting points on PZA-40-Apt biosensor obtained by the two methods, it can be seen that the SERS signal generated by the “dropping” method can clearly identify the distinctive Raman peaks of *S. typhimurium*. In contrast, the SERS signal generated by the “soaking” method is almost negligible. This phenomenon can be explained by the following reason. Due to the superhydrophobicity of the PZA-40-Apt biosensor surface, the orange juice–*S. typhimurium* complex formed by the “dropping” method creates a large contact angle on the biosensor surface. This causes the droplets to retain their original shape instead of spreading over the entire sensor surface to repel the liquid sample. Meanwhile, this superhydrophobic substrate can concentrate the analyte over a small area, hindering the coffee-ring effect and thus achieving significant SERS enhancement [72]. In conclusion, PZA-40-Apt biosensors have great potential as a SERS substrate to detect *S. typhimurium* in practical applications.

### 2.7. Antibacterial Activity of PZA-40-Apt Biosensor and Inactivation Mechanism 

**Figure 8:**
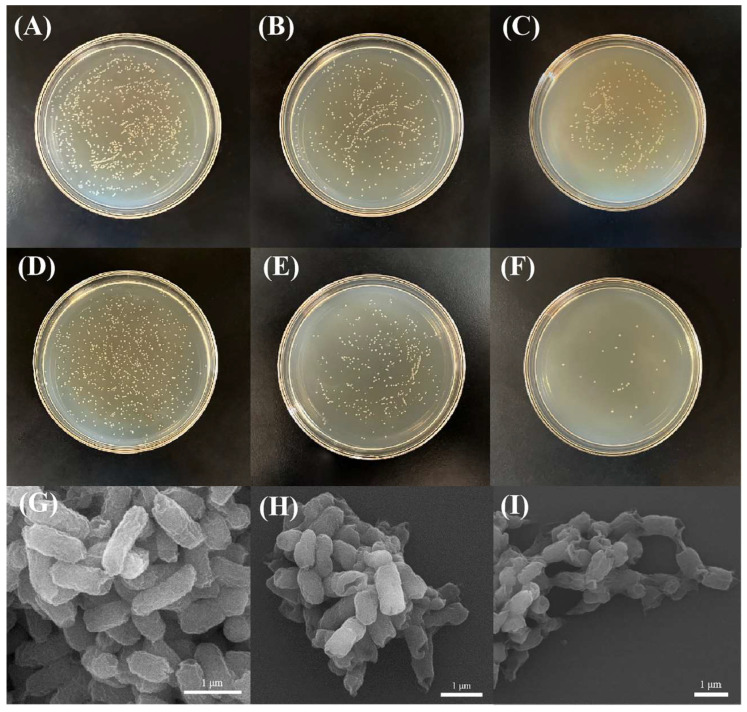
Colony formation assay for *S. typhimurium* culture treated with various samples for 10 min: (**A**) PDMS, (**B**) PDMS-ZnO and (**C**) PZA-40-Apt biosensor under dark conditions; (**D**) PDMS, (**E**) PDMS-ZnO and (**F**) PZA-40-Apt biosensor under visible light irradiation. SEM images of *S. typhimurium* after 10 min of incubation with various samples under visible light irradiation: (**G**) none; (**H**) PDMS-ZnO; (**I**) PZA-40-Apt biosensor.

Inactivating bacteria effectively remains a challenge while achieving ultrasensitive detection of *S. typhimurium*. The antibacterial effectiveness of the PZA-40-Apt biosensor against *S. typhimurium* was assessed using colony formation assay and a SEM test. In comparison to the blank control (Figure S10), the colony number of *S. typhimurium* treated with PDMS for 10 min under dark conditions (Figure 8A) does not significantly change. This indicates that PDMS itself does not possess antibacterial activity against *S. typhimurium*. The colony count of *S. typhimurium* treated with PDMS-ZnO in the dark is significantly lower than that of *S. typhimurium* treated with PDMS (Figure 8B). The antibacterial ability of PDMS-ZnO can be attributed to the existence of Zn^2+^ ions released by partial dissolution of ZnO. The reason is that unbound Zn^2+^ ions have the potential to attach to biofilm surfaces and penetrate into bacteria, whereupon they can interact with sulfhydryl, amino and hydroxyl groups on the bioactive proteases of bacteria, which will interfere with normal metabolism and cause damage to bacterial structure [39,73]. Additionally, the colony number of *S. typhimurium* treated with PZA-40-Apt biosensor under dark conditions is dramatically less than that of *S. typhimurium* treated with PDMS-ZnO (Figure 8C). This can be because the PZA-40-Apt biosensor releases Ag^+^ ions, which have a high bactericidal effect by binding to thiol groups in enzymes on the surface of bacteria [74,75]. Interestingly, after *S. typhimurium* is treated with PDMS under visible light exposure for 10 min, as can be observed in Figure 8D, there is also no discernible change in the colony count of *S. typhimurium* when it is in comparison to the blank control. This indicates that the combination of visible light and PDMS still does not present a distinct antibacterial effect against *S. typhimurium*. By comparison, once *S. typhimurium* treated with PDMS-ZnO is exposed to visible light for 10 min, the colony count is lower, compared to *S. typhimurium* treated with PDMS exposed to visible light (Figure 8E). This is because when visible light is irradiated on the surface of ZnO, a redox reaction takes place to form ROS that can damage the internal bacterial components like DNA, lipids and proteins, as well as the cell membrane. This damage can ultimately result in the death of *S. typhimurium* [76]. In particular, the PZA-40-Apt biosensor produces its strongest bactericidal effect when irradiated with visible light for 10 min, as demonstrated in Figure 8F. This desired sterilization effect is caused by Ag nanocrystals on the ZnO surface. The presence of Ag on the surface of metal oxide semiconductors can significantly improve charge-transfer kinetics at the semiconductor–metal interface. This results in a dual sterilization effect that causes bacterial death by utilizing the synergetic bactericidal effect of both ZnO and Ag nanocrystals [41]. 

The antibacterial efficacy of the PDMS, PDMS-ZnO and PZA-40-Apt biosensor was assessed using the L_b_, as presented in Table 2. In both the dark and the presence of visible light, the L_b_ of *S. typhimurium* treated with PDMS is only 3.5% and 4.2%, respectively. The L_b_ of *S. typhimurium* treated with PDMS-ZnO in the absence and presence of visible light irradiation increases to 38.7% and 53.2%, respectively. By contrast, the L_b_ of *S. typhimurium* treated with PZA-40-Apt biosensor in the absence and presence of visible light irradiation dramatically reaches 65.2% and 97%, respectively.

SEM images of *S. typhimurium* after 10 min of incubation with various samples under exposure to visible light were utilized to verify the antibacterial effect of PDMS-ZnO and the PZA-40-Apt biosensor against *S. typhimurium.* According to Figure 8G, the untreated *S. typhimurium* bacteria with 400 nm diameter and 1.5 µm length exhibit a typical rod-shaped morphology with an elliptical end. However, after S. typhimurium bacteria are treated with PDMS-ZnO under visible light irradiation for 10 min, there are dents at the edges of some bacterial cells, as presented in Figure 8H. The reason is that the cell membrane ruptures and the cell walls collapse, resulting in the leakage of the cytoplasm and finally the death of *S. typhimurium* bacteria [77]. In comparison, as shown in Figure 8I, after *S. typhimurium* is treated with the PZA-40-Apt biosensor, the deformation of bacteria becomes more apparent. Almost all of the *S. typhimurium* bacteria are broken and deformed. In conclusion, the results of the colony formation assay and the SEM further provide more visual evidence for the superior antibacterial action of the PZA-40-Apt biosensor.

The inactivation mechanism of *S. typhimurium* by PDMS-ZnO and the PZA-40-Apt biosensor under visible light irradiation, respectively, is suggested by the above experimental results. The following two factors contribute to visible-light-driven inactivation for *S. typhimurium* by PDMS-ZnO. On one hand, Zn^2+^ ions released from PDMS-ZnO play a bactericidal role [78]. On the other hand, when PDMS-ZnO is irradiated with visible light, it is possible to excite e^−^ from the valence band (VB) of ZnO to the CB to produce photogenerated e^−^-h^+^ pairs, leaving photogenerated h^+^ in the VB [79]. The photogenerated e^−^ and h^+^ can interact with O_2_ and H_2_O or OH^−^ adsorbed on PDMS-ZnO to generate ·O^2−^ and ·OH, respectively. The detailed reactive processes are listed below:

(1) $$O_{2}+e^{-}\to \cdot O^{2-}$$

(2) $${H_{2}O/\mathrm{OH}}^{-}+h^{+}\to \cdot \mathrm{OH}$$

**Scheme 1:**
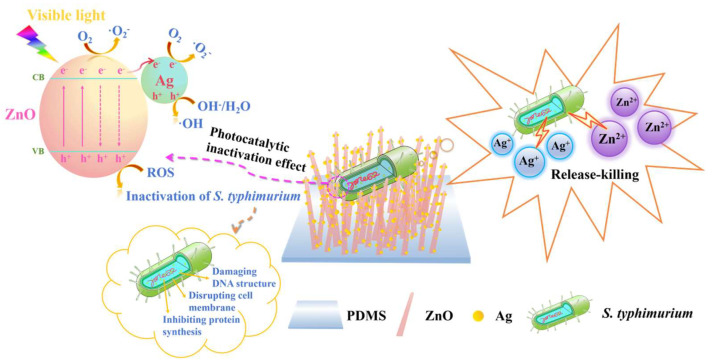
Schematic illustration for synergistic photocatalytic inactivation mechanism of *S. typhimurium* by PZA-40-Apt biosensor under visible light.

ROS including ·O^2−^ and ·OH free radicals generated by the reaction contribute greatly to the inactivation of *S. typhimurium*, which can damage the internal components of the bacteria and ultimately lead to the death of *S. typhimurium* [50,77]. The unique structure of PZA-40-Apt is responsible for its superior antibacterial capabilities over PDMS-ZnO. As depicted in Scheme 1, in addition to the contribution of Zn^2+^ ions and ROS in the killing of *S. typhimurium*, the introduction of Ag nanocrystals forms a Schottky barrier at the metal–semiconductor interface between Ag and ZnO, allowing photogenerated e^−^ to further transfer from the CB of ZnO to Ag, which can significantly restrain the recombination of photogenerated charge carriers [41]. This increases the photocatalytic activity and the carrier lifetime, thereby enhancing the bactericidal effect. Additionally, Ag nanocrystals have a reputation for being potent antibacterial agents against a variety of pathogenic bacteria because they increase the bactericidal activity of PZA-40-Apt biosensors by releasing Ag^+^ ions, which in turn helps to kill *S. typhimurium* [44,48]. 

## 3. Materials and Methods

### 3.1. Chemicals, Biochemicals and Instruments

Information on chemicals, biochemicals and instruments is listed in Supplementary Materials.

### 3.2. Preparation of PDMS-ZnO Films

**Scheme 2:**
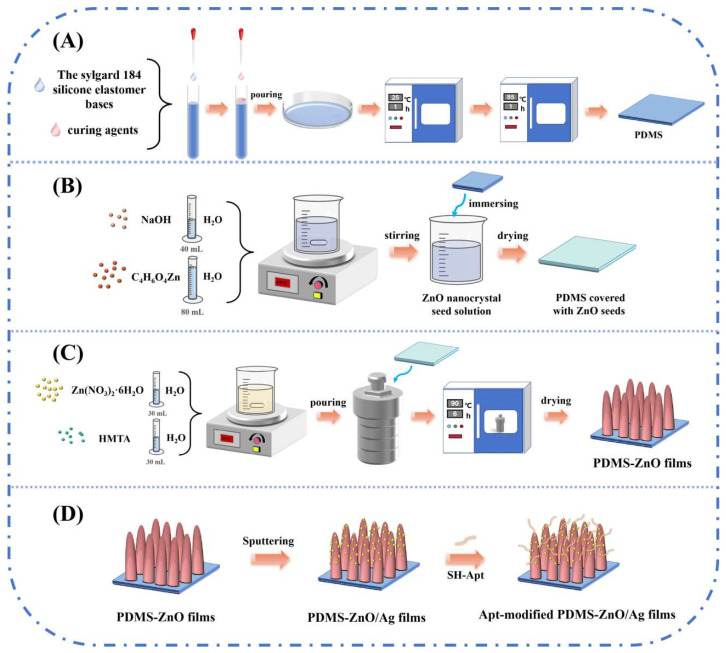
Schematic illustration of the synthesis procedure of (**A**) PDMS, (**B**) PDMS covered with ZnO seeds, (**C**) PDMS-ZnO films and (**D**) Apt-modified PDMS-ZnO/Ag films.

A glass rod was used to thoroughly mix the sylgard 184 silicone elastomer bases and curing agents in a container at a 10:1 weight ratio. The colloid was then poured into the mold and placed in a vacuum, with the aim of removing bubbles. After degassing treatment, the mixture was further cured for 1 h at 85 °C to yield the solidified PDMS film, as presented in Scheme 2A. Subsequently, a simple hydrothermal method was utilized to grow rod-shaped ZnO nanocrystals on PDMS film [80], as presented in Scheme 2B,C. The ZnO nanocrystal seed solution was produced by dropwise adding 40 mL of 0.75 mM NaOH solution to 80 mL of 0.125 mM C_4_H_6_O_4_Zn solution, then stirring at 65 °C for 2 h. To ensure complete coverage of the PDMS surface with the ZnO seed solution, the prepared PDMS film was immersed into the ZnO nanocrystal seed solution for 45 s and then left to dry for 40 min. Meanwhile, a mixture of 30 mL of 60 mM Zn(NO_3_)_2_·6H_2_O and 30 mL of 60 mM HMTA was agitated for 30 min at room temperature. To grow ZnO nanorods on PDMS film, the mixed solution and the PDMS film covered with ZnO seeds were transferred to a Teflon-lined stainless-steel autoclave. After that, the autoclave was heated to 90 °C and held for 6 h, and then it was allowed to cool to room temperature. The resulting PDMS-ZnO films were washed with ultrapure water before being dried at 50 °C for future usage.

### 3.3. Preparation of PDMS-ZnO/Ag Films

Ag nanocrystals were deposited on PDMS-ZnO films using DC magnetron sputtering (ATC 1800F, AJA International Inc., Boston, MA, USA) with Ag target of 99.99% purity. The gas used for sputtering was Ar and the pressure was kept at 5 mTorr. The typical base pressure of the chamber was normally about 3 × 10^−7^ Torr. The power used for Ag deposition was set at around 30 W. The four PDMS-ZnO film samples were named PZA-20, PZA-30, PZA-40 and PZA-50, corresponding to different sputtering times of 20 s, 30 s, 40 s and 50 s, respectively.

### 3.4. SERS Detection of 4-MBA 

To determine the ideal SERS substrate, the influence of Ag sputtering time on SERS signals was investigated. PDMS-ZnO/Ag films with different sputtering times were applied as SERS substrates to detect the reporter molecule (4-MBA). Ten µL of 4-MBA (10^−5^ M) was added dropwise onto the surfaces of PZA-20, PZA-30, PZA-40 and PZA-50, respectively, and then these were dried for subsequent SERS detection. Based on the SERS detection results, PZA-40 exhibited the strongest SERS signal and was therefore identified as the optimal SERS substrate. Subsequently, PZA-40 was used to detect varying concentrations of 4-MBA (10^−3^–10^−10^ M). The source of excitation in this experiment was an Ar^+^ ion laser at 514 nm. The Renishaw inVia Raman system was employed to capture the SERS spectra. The laser power was maintained at 30 mW throughout the SERS spectrum collection. The number of collections was one, the attenuation was 10% and the accumulation time was 10 s.

### 3.5. Preparation of Apt-Modified PDMS-ZnO/Ag Films

TCEP solution was applied to activate the thiolated aptamer (SH-Apt). A quantity of 10 µL of 1 mM TCEP solution was combined with 10 µL of 10^−6^ M SH-Apt. Following a duration of 1 h, 10 µL of the activated SH-Apt solution was dripped onto PDMS-ZnO/Ag films (0.5 cm × 0.5 cm). The Apt-modified PDMS-ZnO/Ag films were rinsed three times with PBS buffer to remove unbound SH-Apt after 24 h incubation at 37 °C. Finally, Apt-modified PDMS-ZnO/Ag films were dried under argon for 6 h and then placed in centrifuge tubes (Scheme 2D). 

### 3.6. Apt-Modified PDMS-ZnO/Ag Films for SERS Detection of S. typhimurium 

In total, 10 µL of *S. typhimurium* with concentrations ranging from 10^1^ to 10^8^ cfu/mL was dripped onto Apt-modified PDMS-ZnO/Ag films. After drying, the films were placed on a SERS detection slide for SERS detection [81,82]. Parameter settings were consistent with Section 3.4 throughout the SERS spectrum collection process.

### 3.7. Antibacterial Test 

The antibacterial efficacy against *S. typhimurium* of the samples was assessed by means of a colony formation assay and SEM. PBS buffer was used to dilute the *S. typhimurium* culture to 10^5^ cfu/mL. The blank control was then prepared by spreading diluted bacterial solution (10 µL) evenly on LB agar plates. The diluted bacterial solutions were then incubated with the samples (PDMS, PDMS-ZnO and Apt-modified PDMS-ZnO/Ag films (0.5 cm × 0.5 cm)), respectively. For the purposes of comparison, three different samples incubated with the bacterial solution were subsequently subjected to dark conditions and exposed to visible light ranging of 420–780 nm for 10 min. Afterwards, 10 µL of bacterial solution was collected in the bacterial solution after incubation with the samples and spread evenly onto LB agar plate. Following a 12 h incubation period at 37 °C, the quantity of colonies was counted. The definition of the bacterial lethality rate (L_b_) was L_b_ = (L_1_ − L_2_)/L_1_ × 100%. Here, L_1_ and L_2_ stand for the colony numbers of *S. typhimurium* that were treated differently and the blank control, respectively.

## 4. Conclusions

In conclusion, a novel multifunctional Apt-modified PDMS-ZnO/Ag biosensor has been demonstrated to be appropriate for SERS detection and photocatalytic sterilization for *S. typhimurium*. The surface Ag content of the PDMS-ZnO/Ag film was changed and 4-MBA was selected as the reporter molecule in order to study the impact of Ag sputtering times with respect to SERS signal intensity. The results indicated that, depending on the quantity of hot spots and photoexcited electrons, the SERS intensity of 4-MBA initially grew and subsequently declined with increasing Ag sputtering time. The logarithmic concentration of *S. typhimurium* was proven to have a highly linear relationship with SERS intensity, ranging from 10^1^ to 10^8^ cfu/mL (y = 1114.65 x − 1001.19, R^2^ = 0.990), and the LOD of *S. typhimurium* detected by the PZA-40-Apt biosensor was 10 cfu/mL. The PZA-40-Apt biosensor demonstrated superhydrophobicity with WCA of as high as 154.5° and could be utilized to detect *S. typhimurium* in freshly squeezed orange juice samples. In addition, the biosensor could efficiently inactivate *S. typhimurium* within 10 min when exposed to visible light, with a L_b_ up to 97%. Thus, effective killing of *S. typhimurium* was achieved. This work provides an exciting new biosensor for identification and treatment of foodborne pathogens, which could have practical applications in the diagnosis of, and therapy for, various clinical conditions.

## Figures and Tables

**Table 1:** Comparison of LOD for 4-MBA between PZA-40 and other SERS substrates.

Detection Method	SERS Substrates	LOD (M)	References
SERS	SiO_2_@Au@Ag NPs	0.74 × 10^−6^	[63]
SERS	Fe_3_O_4_/Ag microspheres	10^−8^	[64]
SERS	Cu_2_O-CuO/Ag	10^−6^	[65]
SERS	Au@Ag NRs	10^−8^	[66]
SERS	PZA-40	10^−9^	this work

**Table 2:** Antibacterial efficacy of PDMS, PDMS-ZnO and PZA-40-Apt biosensor under various conditions.

Sample	Light Condition	Average L_b_ for *S. typhimurium*
PDMS	In the dark	3.5%
PDMS	Under visible light irradiation	4.2%
PDMS-ZnO	In the dark	38.7%
PDMS-ZnO	Under visible light irradiation	53.2%
PZA-40-Apt	In the dark	65.2%
PZA-40-Apt	Under visible light irradiation	97%

## Data Availability

All relevant data are within the paper.

## Supplementary Materials

The following supporting information can be downloaded at: https://www.mdpi.com/article/10.3390/ijms25147675/s1.
