# Supplementary material for: Apt-Conjugated PDMS-ZnO/Ag-Based Multifunctional Integrated Superhydrophobic Biosensor with High SERS Activity and Photocatalytic Sterilization Performance
Source: Int J Mol Sci. 2024 Jul 12;25(14):7675. doi: 10.3390/ijms25147675 (PMC11276906; doi:10.3390/ijms25147675)
Supplement: Supplementary file 1 [file ijms-25-07675-s001.zip › ijms-3083958-supplementary.pdf]

International Journal of *Molecular Sciences*

MDPI

Supplementary Materials

Apt-conjugated PDMS-ZnO/Ag-based multifunctional integrated superhydrophobic biosensor with high SERS activity and photocatalytic sterilization performance

Sihan Qian [1], Wenshi Zhao [1], Rui Guo [1], Xiaohan Wang [1], Huasong Dai [1], Jihui Lang [1], Naveen Reddy Kadasala [2], Yuhong Jiang [1,*] and Yang Liu [1,*]

Chemicals

The chemicals used in the experiment were as follows. Polydimethylsiloxane (PDMS) Sylgard 184 elastomer was obtained from Dow Corning Co., Ltd. (Midland, Mi, USA). Zinc nitrate hexahydrate ($Zn(NO_3)_2{\cdot}6H_2O$) was purchased from Tianjin Damao Chemical Reagent Factory (Tianjin, China). Hexamethylenetetramine (HMTA) and sodium hydroxide (NaOH) were obtained from Tianjin Guangfu Technology Development Co., Ltd. (Tianjin, China) and Shanghai Macklin Biochemical Co., Ltd. (Shanghai, China), respectively. Zinc acetate ($C_4H_6O_4Zn$), 4-mercaptobenzoic acid (4-MBA) and absolute ethanol were procured from Sinopharm Chemical Reagent Co., Ltd. (Beijing, China). Tris-(2-carboxyethyl) phosphine hydrochloride (TCEP), 2.5% glutaraldehyde and phosphate buffer solution (10 mM PBS, pH 7.4) were bought from Aladdin Reagent Co., Ltd. (Shanghai, China). The silver metal target (99.99% purity) was purchased from Beijing Jingmai Zhongke Material Technology Co., Ltd (Beijing, China). All reagents used in this work were of analytical grade and utilized without further purification. All aqueous solutions were synthesized with deionized water with a resistance of 18.2 MΩ·cm purified by a Milli-Q Ultrapure water purification system (Millipore Corp., Bedford, MA, USA). All glassware were first treated with aqua regia for a duration of 24 h and then rinsed with ultrapure water.

Biochemicals

S. typhimurium (CMCC 50115) shock-frozen strain was obtained from Ning Bo testobio Co., Ltd. (Ningbo, Zhejiang, China). The aptamers that could specifically recognize *S. typhimurium* were synthesized by Shanghai Sangon Biological Science & Technology Company (Shanghai, China), and the sequence was 5′-SH-GGG AGC TCA GAA TAA ACG CTC AAG GGC AGG TGT TAT GTG TAC TGC TAC AGT GTG GTT GTT CGA CAT GAG GCC CGG AC-3′ [1].

Instruments

Scanning electron microscopy (SEM) and energy dispersive spectroscopy (EDS) were performed on a scanning electron microscope (JEOL JSM-7800F) to characterize the morphologies and elemental compositions of the prepared samples. The structure of samples was determined by X-ray diffractometer (XRD; Rigaku D/Max-2500) and X-ray photoelectron spectroscopy (XPS; Thermo Scientific ESCALAB 250Xi). The ultraviolet-visible (UV-vis) spectra were recorded on a UV-3600 UV–Vis-NIR spectrophotometer (Shimadzu Co., Kyoto, Japan). The SERS spectra were collected in the region from 400 to 1800 cm^{-1} by means of a Renishaw inVia Raman microscope with 514 nm of excitation (Renishaw, London, UK). The total acquisition time for each SERS spectrum is 10 s. The water contact angles (WCAs) were measured using a contact angle measuring system (OCA 20, Dataphysics, Germany).

Estimation of the enhancement factor

The SERS enhancement factor (EF) was calculated using the following expression:

$$EF = \frac{I_{SERS}}{I_{Bulk}} \times \frac{N_{Bulk}}{N_{SERS}} = \frac{I_{SERS}}{I_{Bulk}} \times \frac{\rho \times h \times S_{laser} \times N_A / M}{f \times S_{laser} / A_{4\text{-}MBA}} \qquad (s1)$$

where I_{SERS} and I_{bulk} are the SERS intensities in SERS spectrum and bulk Raman spectrum of 4-MBA molecules; N_{bulk} and N_{SERS} are the number of 4-MBA molecules under the laser illumination for

the bulk and SERS experiments, respectively. S_{laser}, ρ, M, h and N_A are the laser spot size of 1 μm, the density of 4-MBA molecules of 1.5 g/cm^3, the molar mass of 4-MBA (154.19 g/mol), the effective layer depth of 19 μm and the Avogadro constant (6.02×10^{23}/mol), respectively. The f is the occupation factor for assumed monolayer of 4-MBA molecules (0.5), and A is the footprint of the 4-MBA molecules (~0.54 nm^2). The in-plane at 1588 cm^{-1} was chosen for calculation of the enhancement factor. By substituting the corresponding values into Eq. (1), EF of PZA-40 is estimated to be about 3.3×10^5, while the EF values for PDMS-Ag and PDMS-ZnO are 1×10^5 and 6×10^3, respectively.

Finite-difference time-domain (FDTD) calculations

The spatial distribution of electromagnetic field surrounding PDMS-ZnO/Ag films (PZA-20, PZA-30, PZA-40, and PZA-50) was simulated by using FDTD method. The measurement results shown in SEM images were used to determine the geometrical parameters for structures within FDTD simulation. FDTD calculations employed plane polarized light with a wavelength of 632.8 nm.

Preparation of *S. typhimurium* samples

4 g of Luria-Bertani (LB) agar medium (powder) was precisely weighed. It was dissolved in 100 mL ultrapure water. The resulting mixture was subjected to boiling process until the agar was completely dissolved and then the process was stopped. The culture medium and petri dishes were sterilized for 20 min in the autoclave at 120 °C. After cooling to 55 °C, the culture medium was transferred into petri dishes. Once the culture medium has cooled to room temperature, *S. typhimurium* strain stored at -20 °C was taken out and put onto the culture medium. After that, the strain

was incubated overnight at a constant temperature to allow it to grow. At 600 nm, the optical density (OD) was determined to calculate bacterial concentration (OD_{600}=1 is about 1×10^8 cfu/mL) [2].

Pretreatment process of *S. typhimurium* before SEM analysis

Centrifugation was used to harvested the bacteria and PBS buffer with a pH value of 7.4 was used to wash the bacteria three times. After being fixed for 12 h with 2.5% glutaraldehyde, the bacteria underwent a 10 min dehydration period using a series of ethanol concentrations (30%, 50%, 70%, 85% and 100%). In order to do SEM measurements, the final step involved dropping the solution of bacterial to the silicon wafer.

Figure S1

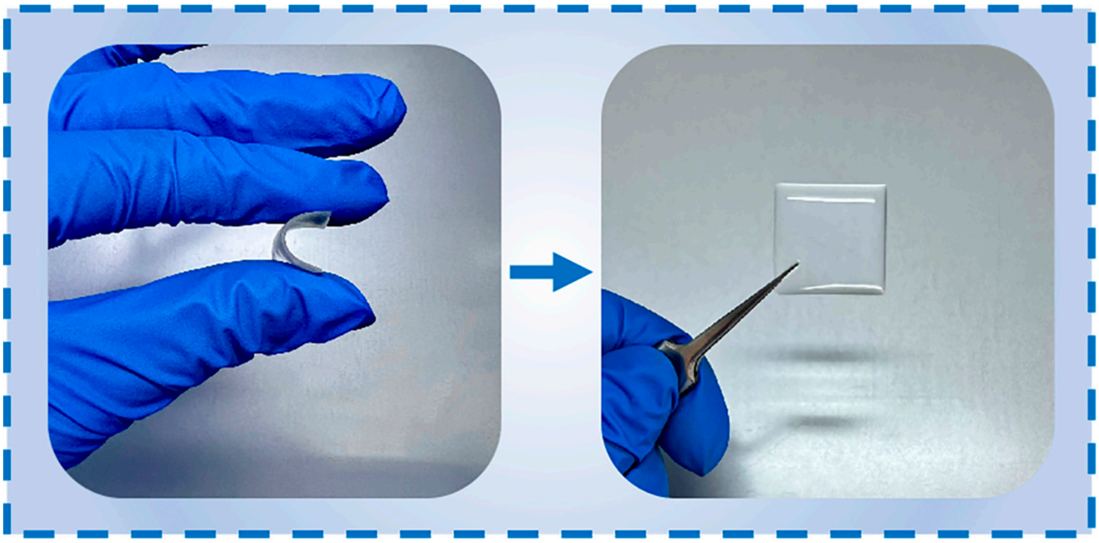

Figure S1. Optical photograph of PDMS after bending and recovering.

Figure S2

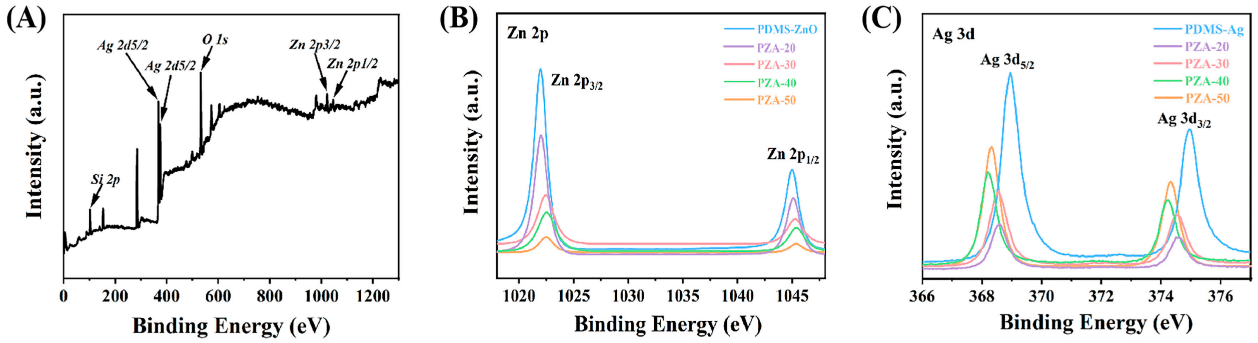

Figure S2. (A) XPS full survey spectrum of PZA-40. (B) High-resolution XPS spectra of Zn 2p of PDMS-ZnO, PZA-20, PZA-30, PZA-40 and PZA-50. (C) High-resolution XPS spectra of Ag 3d obtained from PDMS-Ag, PZA-20, PZA-30, PZA-40 and PZA-50.

Figure S3 82

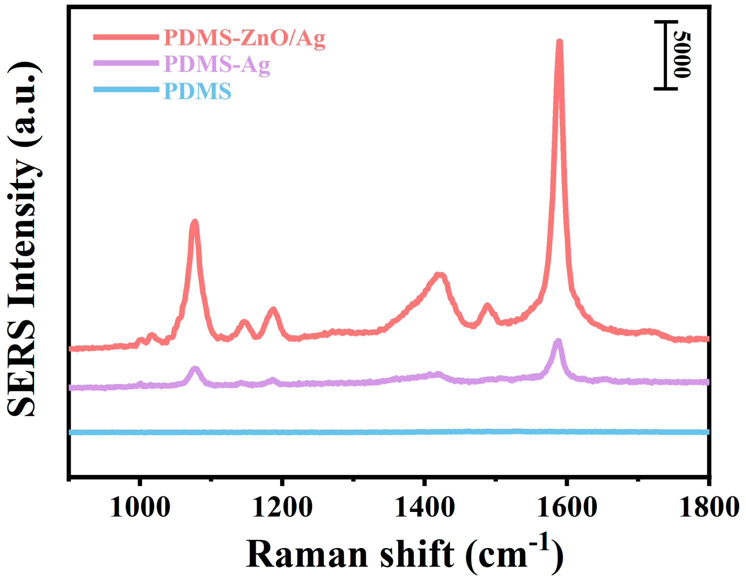

83

Figure S3. SERS spectra of 4-MBA (10^{-5} M) on PDMS, PDMS-Ag and PDMS-ZnO/Ag. 84

85

Figure S4 86

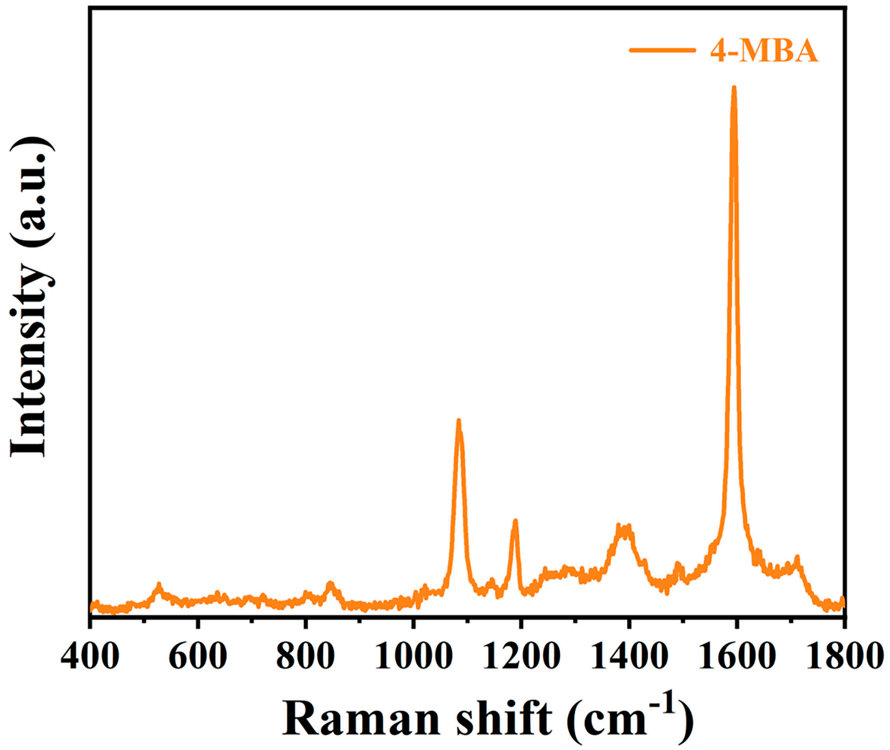

87

Figure S4. Raman spectrum of pure 4-MBA molecules. 88

Figure S5 89

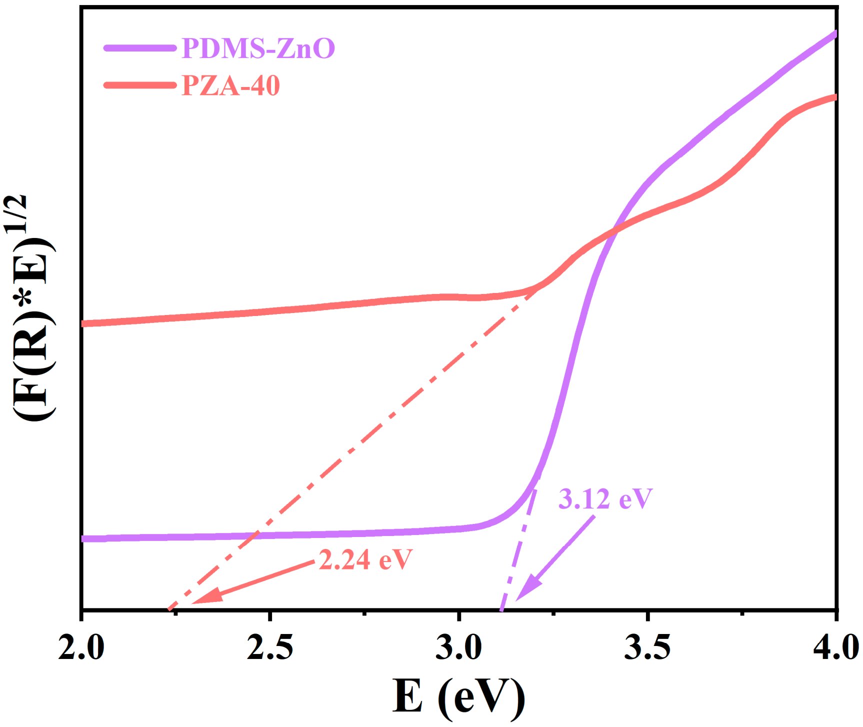

90

Figure S5. Tauc plots of PDMS-ZnO and PZA-40. 91

Figure S6 92

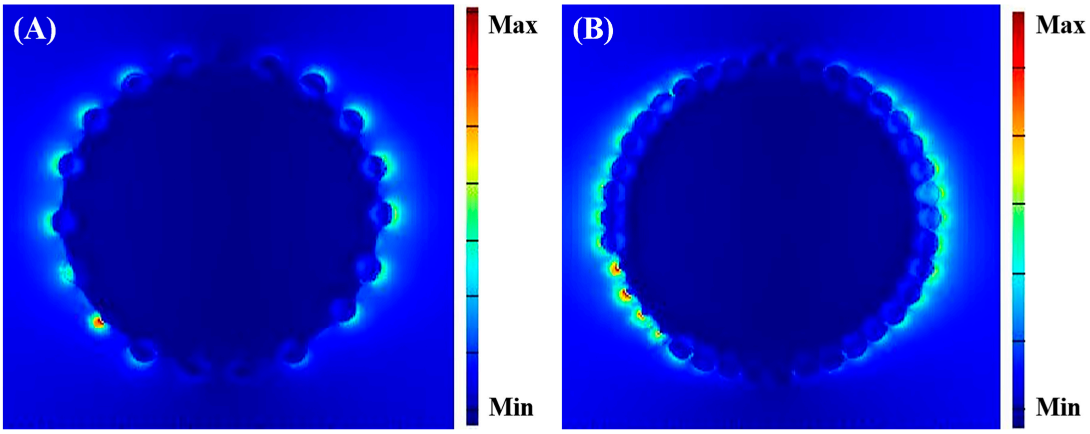

93

Figure S6. Electric field distribution of (A) PZA-30 and (B) PZA-50 simulated by FDTD method. 94

Figure S7 95

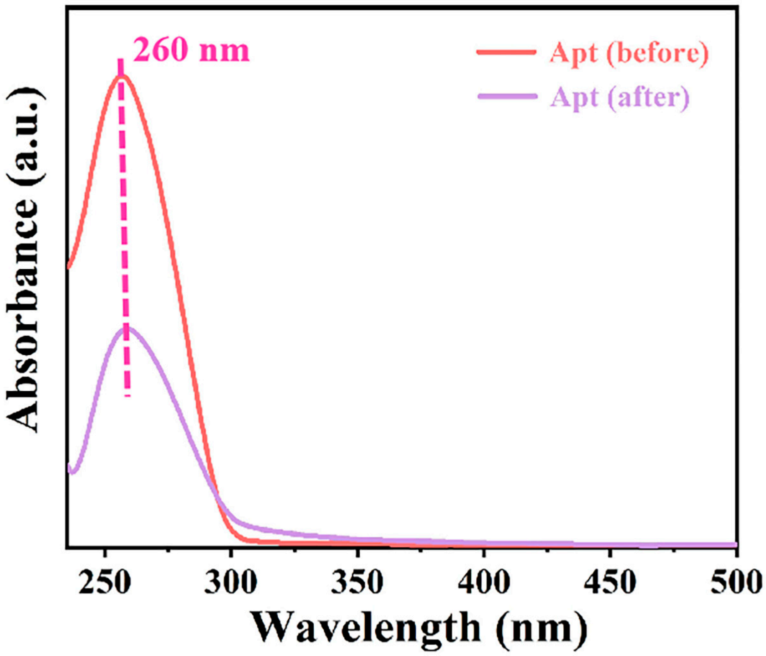

96

Figure S7. UV-Vis absorption spectra of supernatants before (red curve) and after (purple curve) 97
modification PZA-40 with aptamers. 98

Figure S8

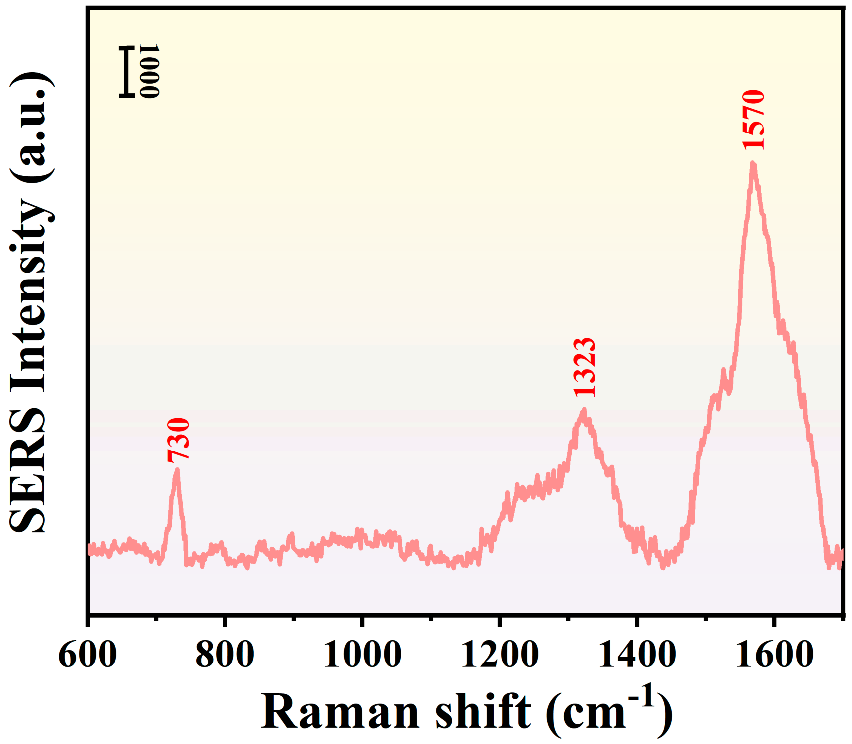

Figure S8. SERS spectrum of *S. typhimurium* at a concentration of 10^7 cfu/mL based on PZA-40-Apt biosensor.

Figure S9

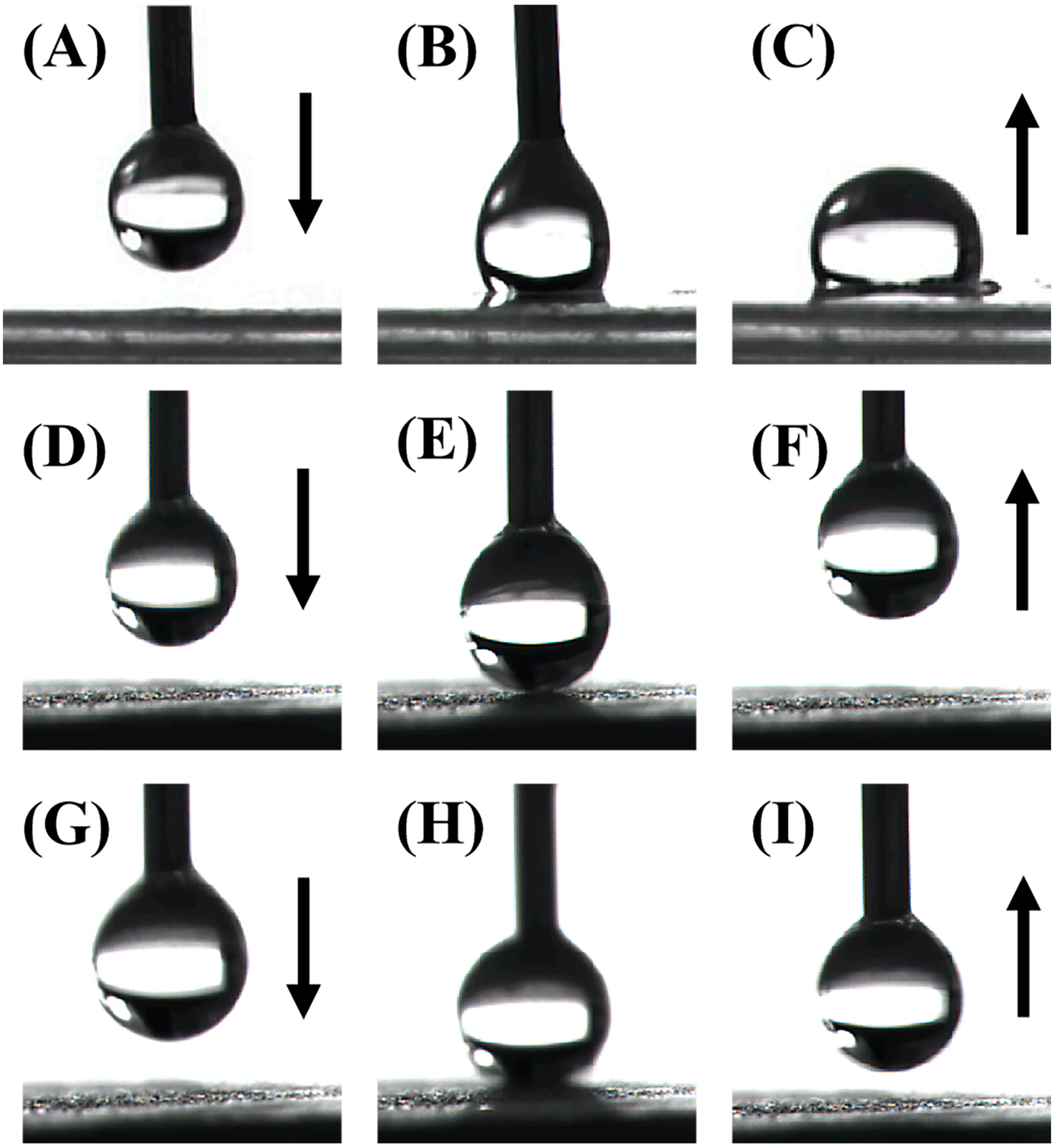

Figure S9. The interfacial behaviors between water droplet and various PDMS substrates: (A-C) PDMS, (D-F) PDMS-ZnO and (G-I) PZA-40-Apt biosensor.

Figure S10

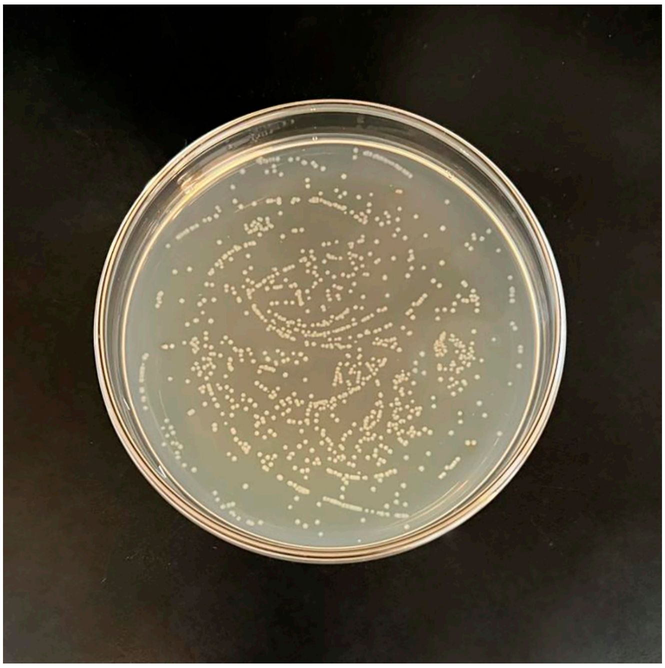

Figure S10. Colony formation assay of the blank control.

Table S1. Band assignments in the SERS spectra of 4-MBA molecules.

Wavenumber (cm^{-1})	Band assignments*	Species
1013	In-plane ring breathing	b_2
1078	In-plane ring breathing + ν(CS)	a_1
1145	C–H deformation modes	b_2
1185	C–H deformation modes	a_1
1421	β(OH) + ν(C-ph) + in-plane ν(CC) + asymmetry ν(CO_2)	b_2
1487	ν(CC) + γ(CH)	
1588	Totally symmetric ν(CC)	a_1

ν stretching, β bending. *For ring vibrations, the corresponding vibrational modes of benzene and the symmetric species under C_{2v} symmetry are indicated.

References

1. Duan, Y.F.; Ning, Y.; Song, Y.; Deng, L. Fluorescent Aptasensor for the Determination of *Salmonella Typhimurium* Based on a Graphene Oxide Platform. *Microchim. Acta* **2014**, *181*, 647-653, doi:10.1007/s00604-014-1170-4.
2. Xu, X.; Ma, X.; Wang, H.; Wang, Z. Aptamer Based SERS Detection of *Salmonella Typhimurium* Using DNA-Assembled Gold Nanodimers. *Microchim. Acta* **2018**, *185*, 325, doi:10.1007/s00604-018-2852-0.
